# Precise Calibration of a GNSS Antenna Array for Adaptive Beamforming Applications

**DOI:** 10.3390/s140609669

**Published:** 2014-05-30

**Authors:** Saeed Daneshmand, Negin Sokhandan, Mohammad Zaeri-Amirani, Gérard Lachapelle

**Affiliations:** Department of Geomatics Engineering, University of Calgary, Schulich School of Engineering, Position Location and Navigation (PLAN) Group, 2500 University Drive, N.W., Calgary, AB T2N 1N4, Canada; E-Mails: nsokhand@ucalgary.ca (N.S.); mzaeriam@ucalgary.ca (M.Z.-A.); gerard.lachapelle@ucalgary.ca (G.L.)

**Keywords:** global navigation satellite system (GNSS), adaptive antenna array beamforming, antenna array calibration, multi-antenna GNSS receiver

## Abstract

The use of global navigation satellite system (GNSS) antenna arrays for applications such as interference counter-measure, attitude determination and signal-to-noise ratio (SNR) enhancement is attracting significant attention. However, precise antenna array calibration remains a major challenge. This paper proposes a new method for calibrating a GNSS antenna array using live signals and an inertial measurement unit (IMU). Moreover, a second method that employs the calibration results for the estimation of steering vectors is also proposed. These two methods are applied to the receiver in two modes, namely calibration and operation. In the calibration mode, a two-stage optimization for precise calibration is used; in the first stage, constant uncertainties are estimated while in the second stage, the dependency of each antenna element gain and phase patterns to the received signal direction of arrival (DOA) is considered for refined calibration. In the operation mode, a low-complexity iterative and fast-converging method is applied to estimate the satellite signal steering vectors using the calibration results. This makes the technique suitable for real-time applications employing a precisely calibrated antenna array. The proposed calibration method is applied to GPS signals to verify its applicability and assess its performance. Furthermore, the data set is used to evaluate the proposed iterative method in the receiver operation mode for two different applications, namely attitude determination and SNR enhancement.

## 1. Introduction

Global navigation satellite system (GNSS) applications utilizing antenna arrays are starting to gain significant attention due to their lower hardware and computational costs. Rapid advances in electronic systems, software define radios (SDR) and miniaturization of radio frequency (RF) front-ends and antenna arrays have opened the way for array processing to play an important role in future receivers [1,2]. Anti-jamming and anti-spoofing methods based on antenna arrays are especially effective for both narrow and wideband interference mitigation and, together with signal to noise ratio (SNR) enhancement, make antenna array processing important for civilian and military applications [3–5].

In general, GNSS applications based on antenna array processing utilize calibrated and non-calibrated array approaches. Methods not requiring calibration do not employ any information about the array configuration, orientation or direction of the incident signals. They do not generally involve the directions of arrival (DOA) of signals in their structure and could be employed as stand-alone methods for anti-jamming and anti-spoofing [5,6]. Although these methods are not complicated in terms of implementation and complexity, they may degrade receiver performance by accidentally nullifying signals. However, in the latter group, the spatial features of GNSS signals are also utilized in the processing. In these methods, the incident directions of the signals are considered in shaping the array beam pattern to not only nullify the undesired signals but also to enhance the SNR of the received signals [7,8]. However, this initially requires an accurately calibrated array to specify the signal DOA, which is referred to as the steering vector or array manifold vector in the literature.

Antenna array calibration has been studied for decades and is employed in many applications such as sonar, radar and direction finding [9,10]. The main array uncertainties that must be considered for a full calibration include uneven cable lengths, array configuration and platform orientation perturbations, unknown antenna element phase center variations, coupling between elements, cross talk in the RF front-ends, antenna gain and phase pattern characteristics and the effect of platform scattering on these patterns. Perturbations in antenna elements positions can occur due to turbulence or tilt of the platform during operation or set up preparation. Even a small inclination error of the platform can degrade the performance of calibration and consequently the spatial processing afterwards. Mutual coupling is the behavior of the coupling between the antennas according to the array configurations and becomes more significant in patch antenna arrays that are not properly isolated. The mutual coupling between antennas is generally proportional to the distances between them and depends on the relative antenna geometry. Phase centre variation is the other consideration that should be taken into account in the calibration procedure unless high quality antenna elements are used in which case phase centres remain constant within 1–2 mm. Although individual antenna elements may have identical horizontal and vertical gain patterns, these will possibly change when assembled into an array due to mutual coupling and scattering. Each of these uncertainties causes an estimated steering vector to deviate from the theoretical one as a function of relative distances between antenna elements and the signal DOA. It is worth mentioning that these uncertainties do not have to be treated separately since most of them are related to each other and affect the array beam pattern in the same manner.

Calibration is traditionally conducted in an anechoic RF chamber such that the antenna array receives a signal from a precisely known direction. The signal direction is changed in incremental steps until all directions of interest are included. Amplitudes and phases are then measured in the receiver as a function of the incident signal direction. The calibration methods proposed in the literature generally differ in modeling and estimating the uncertainties. GNSS array calibration can also be carried out with live line of sight signals since the satellite positions are known. Antenna calibration using GNSS signals and resulting challenges such as mutual coupling, phase centre variations, cross talk in the RF-front end and other effects have recently been studied in the literature [11–15].

This paper first derives a new model taking all of the factors that produce calibration uncertainties into account. This model separates the constant unknown parameters and those that are dependent on signals DOA to simplify the interpretation of the calibration uncertainties and, more importantly, enable the use of the calibration results for steering vector estimation. A two-stage optimization is employed to fit the measurements with the proposed model in which the first stage estimates the constant unknown parameters and the second stage finds the DOA dependent part and save them in the form of a set of look up tables, each of which assigns amplitude and phase compensation coefficients to one antenna element.

In the next part of the paper, the second mode, which is the receiver operation mode, is described. During the calibration procedure, accuracy is the main concern rather than processing time and computational complexity since the calibration procedure for a fixed antenna array platform generally needs to be performed only once. However in applying the calibration results in the receiver operation mode, the processing time and computation complexity are important concerns. For this reason, in the operation mode, an iterative method with a very fast convergence is proposed to estimate the steering vectors of the satellite signals by applying the estimated calibration coefficients and matching the compensation phase and amplitude coefficients from prepared look up tables.

In order to evaluate the performance and effectiveness of both methods introduced above, a set of real GPS L1 signals was collected and processed. The experimental set up was similar to [15] where a tactical grade IMU, to provide the orientation angles (pitch, roll, and heading) and an antenna array were mounted on a moving vehicle collecting GPS signals. In [15], an effective approach was proposed in which a certain number of virtual antennas was considered to take potential phase centre variations of 1 cm into account. For this purpose, a least squares algorithm was used to find the best fit for all measurements without assigning any explicit model to the uncertainties. In this approach, the virtual antennas were positioned at a distance of 1 cm from each physical antenna in each direction in the body frame coordinate system. This may reduce the accuracy of calibration since only the phase centre variations in certain positions and directions were considered.

In Section 2 the system model is presented. Section 3 introduces the two-stage optimization for calibration, followed by proposing the iterative method for estimating satellite steering vectors. Practical tests and data analyses are provided in Section 4 and finally, Section 5 concludes the paper.

*Notation*: Throughout this paper, the following notation is adopted: small bold letters stand for vectors and capital bold letters stand for matrices. Superscripts *H* and *T* denote conjugate transpose and transpose, respectively. Function *vec*(**A**) denotes the vectorization operator obtained by stacking the rows of the matrix **A** on top of one another. Function *diagMat*(**A**) returns a column vector of the main diagonal elements of **A** and function *diagVec*(**a**) returns a square diagonal matrix with the elements of **a**. (**A**)^−1^ and (**A**)^#^ symbolize the inverse and pseudoinverse of matrix **A**, respectively. ‖**a**‖ denotes the Euclidean norm of vector **a**. |**a**| and ∠**a** denote vectors containing the amplitude and phase of the elements of **a**. Finally, **I***_N_* and **0***_N_*_×_*_M_* represent the *N*×*N* identity matrix and *N*×*M* all-zero matrix, respectively.

## 2. System Model

**Figure 1.**
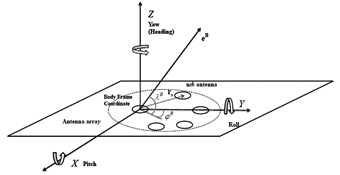
Antenna array configuration in the body frame coordinate system.

Assume an *N*-element antenna array with an arbitrary configuration placed on a platform which is fixed with respect to the body frame coordinate system. In this configuration, one antenna is chosen as a reference antenna. For the sake of simplicity, it is assumed that the origin of the body frame coordinate system is located at the reference antenna location shown in Figure 1.

Under the assumption of perfect antenna elements (identical isotropic elements) and ignoring the measurement noise, the *N*×1 complex baseband signal vector consisting of measured phases and amplitudes at different antennas received from one satellite can be written as:

(1) $$\text{y}\;=\text{Ca}e^{j\theta (t)}$$

where *θ*(*t*) considers the phase changes due to the Doppler frequency variation, which is a function of time due to both satellite and vehicle motions and **a** is an *N*×1 vector representing the steering vector of the satellite signal defined as [16]:

(2) $$\text{a}\;≜\left[\begin{array}{c} 1\\ e^{j\frac{2\pi }{\kappa }{\left({\text{e}}^{B}\right)}^{T}{\text{r}}_{2}}\\ \vdots \\ e^{j\frac{2\pi }{\kappa }{\left({\text{e}}^{B}\right)}^{T}{\text{r}}_{N}} \end{array}\right]$$

in which *κ* is the wavelength of the signal and **r***_n_*, *n*=2,…,*N* is a 3×1 vector pointing to the *n*^th^ antenna element and **e***^B^* is a 3×1 unit vector pointing to the satellite in the body frame coordinate system. Without loss of generality, the first antenna is chosen as the reference antenna where the coordinate system origin is located. In Equation (1), **C** is a *N*×*N* matrix representing constant uncertainties due to any imperfection from antennas to digitizers. The diagonal elements of **C** model the difference in amplitudes and phases due to unequal cable lengths and other electronic parts and its off-diagonal elements represent the coupling coefficients between antennas. Nevertheless this model is not yet sufficiently accurate since the antenna elements are not isotropic and they have different gain and phase radiation patterns. Moreover, phase center variations and uncertainties of the array configuration should be also taken into the account to increase the accuracy of the calibration process. To include these variant uncertainties, **y** should be re-expressed as:

(3) $$\text{y}\;=\text{CP}(\varphi^{B},\lambda^{B})\text{a}e^{j\theta (t)}$$

where **P**(*φ^B^*, *λ^B^*) is a *N*×*N* matrix defined as:

(4) $$\begin{array}{l} \text{P}(\varphi^{B},\lambda^{B})\\ ≜\left[\begin{array}{cccc} a_{1}(\varphi^{B},\lambda^{B})e^{j\omega_{1}(\varphi^{B},\lambda^{B})}e^{j\frac{2\pi }{\kappa }{\left({\text{e}}^{B}\right)}^{T}\Delta {\text{r}}_{1}} & 0 & \cdots & 0\\ 0 & a_{2}(\varphi^{B},\lambda^{B})e^{j\omega_{2}(\varphi^{B},\lambda^{B})}e^{j\frac{2\pi }{\kappa }{\left({\text{e}}^{B}\right)}^{T}\Delta {\text{r}}_{2}} & & 0\\ \vdots & & \ddots & \vdots \\ 0 & 0 & \cdots & a_{N}(\varphi^{B},\lambda^{B})e^{j\omega_{N}(\varphi^{B},\lambda^{B})}e^{j\frac{2\pi }{\kappa }{\left({\text{e}}^{B}\right)}^{T}\Delta {\text{r}}_{N}} \end{array}\right] \end{array}$$

in which Δ**r***_n_* is a 3×1 vector representing the perturbation of the *n*^th^ antenna element position in the body frame coordinate system, and *a _n_*(*φ^B^ , λ^B^*) and *ω _n_*(*φ^B^, λ^B^*) are amplitude and phase deviations of the received signal at elevation *λ^B^* and azimuth *φ^B^* in the body frame coordinate system for the *n*^th^ antenna element. This term considers imperfection of the antenna elements, radiation patterns, phase center variations and any other signal DOA-dependent phenomena.

**Figure 2.**
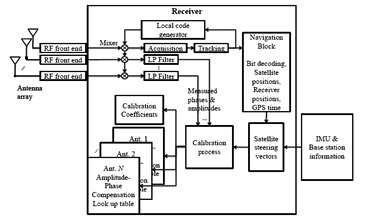
Multi-antenna receiver structure in the calibration mode.

Since the proposed calibration method is based on receiving GNSS signals during antenna platform motion, Doppler frequency varies due to the changes in the relative velocity between the antenna array and the satellites, which are not negligible. Even in the static case, Doppler frequency is gradually changing due to satellite motion. In the calibration process using a multi-antenna receiver, acquisition, tracking and positioning procedures can be only performed for the received satellite signals at the reference antenna (reference antenna) and the generated local codes can be used to despread the received signals at the other antennas (secondary antennas) as shown in Figure 2.

During the tracking stage of the signal of each satellite, the phase of the signal at the reference antenna is kept approximately zero and the phase of the signals at the other antennas are measured relative to that of the reference antenna. For simplicity, amplitudes are also normalized with respect to the signal at the reference antenna. This removes the term responsible for Doppler frequency changes from observations as well as phase changes due to the alteration of signal DOA for the master antenna. Therefore, the normalized signal vector that is actually obtained from the multi-antenna receiver, denoted by, **ỹ** is related to **y** as:

(5) $$\text{ỹ}=\frac{\text{y}}{\delta^{T}\text{CPa}e^{j\theta (t)}}$$

where $\underset{N\times 1}{\delta }={\left[\begin{array}{cccc} 1 & 0 & \cdots & 0 \end{array}\right]}^{T}$ (with the first antenna chosen as the reference antenna). In Equation (4), **P**(*φ^B^ , λ^B^*) is a function of *φ^B^* and *λ^B^* in the body frame coordinate system. However, a GNSS receiver provides azimuth and elevation angles in the geographic coordinate system such as local coordinate system ENU. Azimuth and elevation angles in the ENU coordinate system, symbolized with *φ^ENU^* and *λ^ENU^*, can be related to the *φ^B^* and *λ^B^* as:

(6) $${\text{e}}^{B}={\text{R}}^{T}{\text{e}}^{\text{ENU}}$$

in which **e***^B^* and **e***^ENU^* are unit vectors pointing to the satellite in the body frame and ENU coordinate systems, respectively, which can be expressed as:

(7) $$\begin{array}{l} {\text{e}}^{B}={\left[\begin{array}{ccc} \sin\left(\varphi^{B}\right)\cos\left(\lambda^{B}\right) & \cos\left(\varphi^{B}\right)\cos\left(\lambda^{B}\right) & \sin\left(\lambda^{B}\right) \end{array}\right]}^{T}\\ {\text{e}}^{\text{ENU}}={\left[\begin{array}{ccc} \sin\left(\varphi^{\text{ENU}}\right)\cos\left(\lambda^{\text{ENU}}\right) & \cos\left(\varphi^{\text{ENU}}\right)\cos\left(\lambda^{\text{ENU}}\right) & \sin\left(\lambda^{\text{ENU}}\right) \end{array}\right]}^{T} \end{array}$$

In Equation (6), **R** is a transformation matrix from the ENU coordinate to the body frame coordinate defined as:

(8) $$\text{R}\;=\left[\begin{array}{ccc} \cos\left(y\right)\cos\left(r\right)-\sin\left(r\right)\sin\left(p\right)\sin\left(y\right) & -\sin\left(y\right)\cos\left(p\right) & \cos\left(y\right)\sin\left(r\right)+\sin\left(y\right)\sin\left(p\right)\cos\left(r\right)\\ \sin\left(y\right)\cos\left(r\right)+\sin\left(r\right)\sin\left(p\right)\cos\left(y\right) & \cos\left(y\right)\cos\left(p\right) & \sin\left(y\right)\sin\left(r\right)-\cos\left(y\right)\sin\left(p\right)\cos\left(r\right)\\ -\sin\left(r\right)\cos\left(p\right) & \sin\left(p\right) & \cos\left(p\right)\cos\left(r\right) \end{array}\right]$$

in which *r*, *p* and *y* refer to the roll, pitch and yaw (heading) angles, respectively (shown in Figure 1). In the proposed method, an IMU is providing accurate estimates of these angles.

## 3. Proposed Method

### 3.1. Receiver Calibration Mode

The model proposed in Equation (3) separates the uncertainties into constant and variable parts wherein the variable part is a function of the received signal DOA. In the calibration process, the polar plane formed by *φ^B^* and *λ^B^* is first partitioned into a uniform grid consisting of a set of cells represented by their centre coordinates as {(Φ*_l_*, Λ*_k_*);*l* = 1,⋯,*g_A_*, *k* = 1, ⋯, g*_E_*}, where *g_A_* and *g_E_* specify the resolution of the grid. This provides a trade-off between accuracy and computational complexity. Considering this model, for each cell in the grid, the calibration process can be expressed as the following optimization problem:

(9) $$\underset{\text{C},\text{P}(\Phi_{l},\Lambda_{k})}{\min}\sum_{i}{\left\|{\text{y}}_{i}-\text{CP}(\Phi_{l},\Lambda_{k}){\text{a}}_{i}e^{j\theta (t)}\right\|}^{2},\;\forall i|(\varphi_{i}^{B},\lambda_{i}^{B})\in (\Phi_{l},\Lambda_{k})$$

where index *i* refers to observables from different satellites at different times. For each cell in the grid, only those observables whose azimuth and elevation angles are located at that cell are employed and **P**(Φ*_l_*, Λ*_k_*) is a diagonal matrix representing the DOA-dependent part of the array uncertainty associated to that cell of the grid. Without considering any specific assumption, the unique estimation of **C** and **P**(Φ*_l_*, Λ*_k_*) is not possible. In appendix A, it is proven that *vec*(**CP**(Φ*_l_*, Λ*_k_*)) belongs to a subspace with a dimension of *N*^2^−*N*+1 and therefore any vector in this subspace satisfies the optimization in Equation (9). As one solution for this optimization (**C̄** and **P̄**(Φ*_l_*, Λ*_k_*)), a two-stage process is considered. The first stage finds the best constant matrix **C̄** from all observables in the entire grid and then, considering **C̄**, the second stage obtains the corresponding diagonal compensation matrix **P̄**(Φ*_l_*, Λ*_k_*) for each cell in the grid from the observables corresponding to that cell. This two-stage optimization can be expressed as follows:

(1) In the first stage, it is assumed that there is no dependency on signals DOA, *i.e.*, **P**(Φ*_l_*,Λ*_k_*) = **I***_N_* and only the constant matrix **C̄** is considered. Given Equations (5) and (9), **C̄** can be estimated from the following minimization problem:
  (10) $$\underset{\begin{array}{c} \bar{\text{C}}\\ \left\|\text{vec}(\bar{\text{C}})\right\|=1 \end{array}}{\min}\sum_{i}{\left\|{\text{ỹ}}_{i}\delta^{T}\bar{\text{C}}{\text{a}}_{i}-\bar{\text{C}}{\text{a}}_{i}\right\|}^{2}$$
(2) In the second stage, the estimated **C̄** is substituted in Equation (9) and the corresponding diagonal compensation matrix **P̄**(Φ*_l_*, Λ*_k_*) for each cell in the grid is estimated as:
  (11) $$\underset{\begin{array}{c} \bar{\text{P}}(\Phi_{l},\Lambda_{k})\\ \left\|\text{diagMat}(\bar{\text{P}}(\Phi_{l},\Lambda_{k}))\right\|=1 \end{array}}{\min}\sum_{i}{\left\|{\text{ỹ}}_{i}\delta^{T}\bar{\text{C}}\bar{\text{P}}(\Phi_{l},\Lambda_{k}){\text{a}}_{i}-\bar{\text{C}}\bar{\text{P}}(\Phi_{l},\Lambda_{k}){\text{a}}_{i}\right\|}^{2},\;\forall i|(\varphi_{i}^{B},\lambda_{i}^{B})\in (\Phi_{l},\Lambda_{k})$$

In Equations (10) and (11), constraints avoid trivial solutions that are **C̄** = 0 and **P̄**(Φ*_l_*, Λ*_k_*) = 0. Considering appendix A and after some matrix manipulations, one can verify that the matrices **C̄** and **P̄**(Φ*_l_*, Λ*_k_*) can be estimated from the following eigenvalue decomposition problems as:

(12) $$\begin{array}{lll} (1)\underset{\left\|\text{c}\right\|=1}{\underset{\text{c}}{\min}} & {\text{c}}^{H}\text{Bc} & \\ (2)\underset{\left\|{\text{p}}_{l,k}\right\|=1}{\underset{{\text{p}}_{l,k}}{\min}} & {\text{p}}_{l,k}^{H}{\text{D}}_{l,k}{\text{p}}_{l,k} & \forall i|(\varphi_{i}^{B},\lambda_{i}^{B})\in (\Phi_{l},\Lambda_{k}) \end{array}$$

where:

(13) $$\begin{array}{l} \text{B}\;≜\underset{i}{\text{∑}}{\text{A}}_{i}^{H}{\left({\text{ỹ}}_{i}\delta^{T}-{\text{I}}_{N}\right)}^{H}\left({\text{ỹ}}_{i}\delta^{T}-{\text{I}}_{N}\right){\text{A}}_{i}\\ {\text{D}}_{l,k}≜\underset{i}{\text{∑}}\text{diagVec}{\left({\text{a}}_{i}\right)}^{H}{\bar{\text{C}}}^{H}{\left({\text{ỹ}}_{i}\delta^{T}-{\text{I}}_{N}\right)}^{H}\left({\text{ỹ}}_{i}\delta^{T}-{\text{I}}_{N}\right)\bar{\text{C}}\text{diagVec}({\text{a}}_{i})\\ \text{c}\;≜\text{vec}(\bar{\text{C}})\\ {\text{p}}_{l,k}≜\text{diagMat}(\bar{\text{P}}(\Phi_{l},\Lambda_{k})),\;l=1,\cdots ,g_{A},\;k=1,\cdots ,g_{E} \end{array}$$

and **A***_i_* is *N*×*N*^2^ matrix defined as:

(14) $${\text{A}}_{i}≜\left[\begin{array}{cccc} {\text{a}}_{i}^{T} & 0_{1\times N} & \cdots & 0_{1\times N}\\ 0_{1\times N} & {\text{a}}_{i}^{T} & \cdots & 0_{1\times N}\\ \vdots & \vdots & \ddots & \vdots \\ 0_{1\times N} & 0_{1\times N} & \cdots & {\text{a}}_{i}^{T} \end{array}\right]$$

From Equation (12), **c** and **p***_l,k_* are the eigenvectors corresponding to the smallest eigenvalues of **B** and **D***_l,k_*, respectively. It is straightforward to verify that *vec*(**C̄P̄**(Φ*_l_*, Λ*_k_*)) is one of the solutions of minimization in Equation (9). Applying **C̄** and **P̄**(Φ*_l_*, Λ*_k_*)), *l* = 1,⋯,*g_A_ k* = 1,⋯, g*_E_*, to the received signal vectors leads to a calibrated array and consequently to accurate estimates of satellite steering vectors.

The second stage of the calibration process takes significant time (a few hours is needed to allow the elevation angles of satellites to change in the sky for a full calibration) to extract the DOA-dependent calibration uncertainties. On the other hand, the first stage which compensates for the constant parameters can be performed rapidly (in a few seconds). In many applications, the antenna array configuration is fixed with respect to the body-frame coordinate system and therefore, the radiation pattern of the array, obtained once from the second stage of calibration, does not change. However, the cable lengths, the initial phases of front-end channels and other parameters modeled through the constant matrix **C̄** may change from one operation to another one. In this case, by considering **P̄**(Φ*_l_*, Λ*_k_*) stored in the look up tables, a fast recalibration process can be performed to only reestimate **C̄**.

### 3.2. Receiver Operation Mode

**Figure 3.**
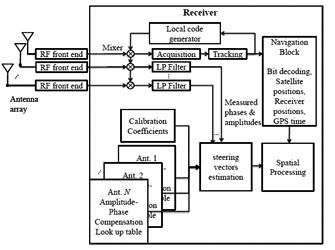
Multi-antenna receiver structure in the operation mode.

Estimation of steering vectors of the incoming signals can be employed in many GNSS multi-antenna applications such as anti-interference, multipath mitigation, SNR enhancement. However, in most cases this cannot be done unless employing a calibrated array. In this section, it is assumed that the antenna array is calibrated using the method introduced in the previous section. In other words, the amplitude and phase compensation coefficients are available through matrix **P̄**(Φ*_l_*, Λ*_k_*), *l* = 1,⋯,*g_A_ k* = 1,⋯,g*_E_*, in the form of several look-up tables, and the constant matrix **C̄**. The block diagram of a multi-antenna receiver in the operation mode is shown in Figure 3. It is worth mentioning that in order to estimate satellite signal steering vectors, position information is only used to calculate the azimuth and elevation angles of incident signals in the ENU coordinate system. These angles can be accurately estimated even with a low cost GNSS receiver (due to the long distance between satellite and the receiver). Therefore, assuming a perfect calibration, position errors do not significantly affect the accuracy of the estimated angles and consequently of the estimated steering vectors.

An iterative approach for estimating the steering vector is now proposed. Considering Equation (3), after calibration, the steering vector can be stated as a normalized solution of a least squares (LS) problem represented by (normalized since amplitude of a steering vector does not convey any information):

(15) $${\text{â}}_{i}=\frac{{\left({\bar{\text{P}}}^{H}(\Phi_{l},\Lambda_{k}){\bar{\text{C}}}^{H}\bar{\text{C}}\bar{\text{P}}(\Phi_{l},\Lambda_{k})\right)}^{-1}{\bar{\text{P}}}^{H}(\Phi_{l},\Lambda_{k}){\bar{\text{C}}}^{H}{\text{ỹ}}_{i}}{\left\|{\left({\bar{\text{P}}}^{H}(\Phi_{l},\Lambda_{k}){\bar{\text{C}}}^{H}\bar{\text{C}}\bar{\text{P}}(\Phi_{l},\Lambda_{k})\right)}^{-1}{\bar{\text{P}}}^{H}(\Phi_{l},\Lambda_{k}){\bar{\text{C}}}^{H}{\text{ỹ}}_{i}\right\|}$$

Although the values of Φ*_l_*,Λ*_k_* are not available at the beginning, these angles are related to the unknown steering vector of interest through Equations (2) and (7). For this reason, herein, an iterative method is suggested to solve Equation (15) through the following steps:

Step (1) Initialize **â***_i_* as ${\text{â}}_{i}(0)=\frac{{\left({\bar{\text{C}}}^{H}\bar{\text{C}}\right)}^{-1}{\bar{\text{C}}}^{H}{\text{ỹ}}_{i}}{\left\|{\left({\bar{\text{C}}}^{H}\bar{\text{C}}\right)}^{-1}{\bar{\text{C}}}^{H}{\text{ỹ}}_{i}\right\|}$, q = 0;
Step (2) At the *q*^th^ recursion, considering Equation (2), **e***^B^* can be derived as:
  (16) $${\text{e}}^{B}=\frac{\kappa }{2\pi }{\left({\text{T}}^{H}\text{T}\right)}^{\#}{\text{T}}^{H}∠{\text{a}}_{i}(\text{q})$$
  in which array configuration matrix **T** ( which is known) is defined as:
  (17) $$\text{T}=\left[\begin{array}{c} {\text{r}}_{1}^{T}\\ {\text{r}}_{2}^{T}\\ \vdots \\ {\text{r}}_{N}^{T} \end{array}\right]$$
Step (3) Considering Equation (7), compute Φ*_l_*(q), Λ*_k_*(q);
Step (4) Use Equation (15) to compute **â***_i_*(q) as follows:
  $${\text{â}}_{i}(\text{q})=\frac{{\left({\bar{\text{P}}}^{H}(\Phi_{l}(\text{q}),\Lambda_{k}(\text{q})){\bar{\text{C}}}^{H}\bar{\text{C}}\bar{\text{P}}(\Phi_{l}(\text{q}),\Lambda_{k}(\text{q}))\right)}^{-1}{\bar{\text{P}}}^{H}(\Phi_{l}(\text{q}),\Lambda_{k}(q)){\bar{\text{C}}}^{H}{\text{ỹ}}_{i}}{\left\|{\left({\bar{\text{P}}}^{H}(\Phi_{l}(\text{q}),\Lambda_{k}(\text{q})){\bar{\text{C}}}^{H}\bar{\text{C}}\bar{\text{P}}(\Phi_{l}(\text{q}),\Lambda_{k}(\text{q}))\right)}^{-1}{\bar{\text{P}}}^{H}(\Phi_{l}(\text{q}),\Lambda_{k}(\text{q})){\bar{\text{C}}}^{H}{\text{ỹ}}_{i}\right\|}$$
Step (5) Set q = q+1 and return to Step 2 (next recursion).

It can be observed that estimating the uncertainties in two stages (the constant part and the compensation look up tables) enhances the robustness of the current steering vector estimation method. In fact, the initialization from the input measurement and constant matrix **C̄** leads to a rough estimate of the steering vector and then, after a few iterations, a proper compensation matrix for an accurate estimate of the steering vector can be obtained. Without this initialization, the method would not necessarily converge. Since **C̄** and **T** are constant matrices and **P̄** is a diagonal matrix, one can easily verify that matrix inverses are not required in the loop. For example (**T***^H^***T**)^#^**T***^H^* and (**C̄***^H^***C̄**)^−1^**C̄***^H^* can be calculated beforehand. These features make the proposed method low-complex and fast-converging, which is suitable for real-time applications employing a precisely calibrated antenna array.

## 4. Testing

### 4.1. Data Collection Setup

A set of GPS L1 signals was collected to test the proposed calibration approach. In this data collection, an array of three antennas (Antcom L1 GPS antennas due to their relatively stable phase centre) mounted on the top of a vehicle was employed. The three antennas, after amplification, were connected to a three-channel National Instrument (NI) RF front-end in which the channels are synchronized through a common clock. The received signals were then sampled, down converted and stored for post processing. One of the antennas was chosen as the reference antenna and the origin of the body frame coordinate system. The received signal at this antenna was split into two branches and the second branch was connected to a NovAtel SPAN™ LCI system (NovAtel Inc., Calgary, AB, Canada), which includes a NovAtel SPAN^®^ enabled GNSS/INS receiver (SPAN SE) and a tactical grade IMU LCI. The low noise and stable biases of the accelerometer and gyro sensors make the IMU well suited for ground or airborne survey applications. IMU measurements were sent to the receiver where a blended GNSS/INS position, velocity and attitude solution was generated at a rate of 200 Hz [17]. Raw GPS data was also collected under LOS (line of sight) conditions using a NovAtel ProPak-V3 receiver as a base station (fixed on the rooftop of a building located on the campus of the University of Calgary) to provide the navigation data bits and differential positioning. The data collected by SPAN LCI system and the base station file were then fed to the NovAtel Inertial Explorer^®^ post-processing software to produce reference positions, velocities and high accurate orientation components.

**Figure 4.**
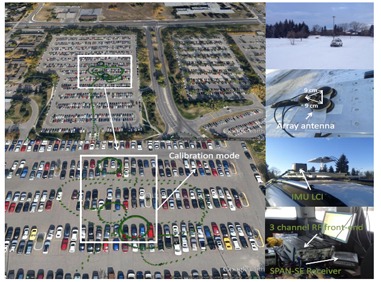
Data collection set up.

The data collection setup and the test trajectory are shown in Figure 4. In order to operate under an open-sky environment, a parking lot was chosen for the data collection. This parking area is almost surrounded by empty fields (low multipath). Circular motion was performed for the calibration process to cover all azimuth angles. In order to cover all possible elevation angles, the circular motion should be repeated during several time intervals with long enough separations to allow satellites to move significantly in the sky.

### 4.2. Calibration Results

**Figure 5.**
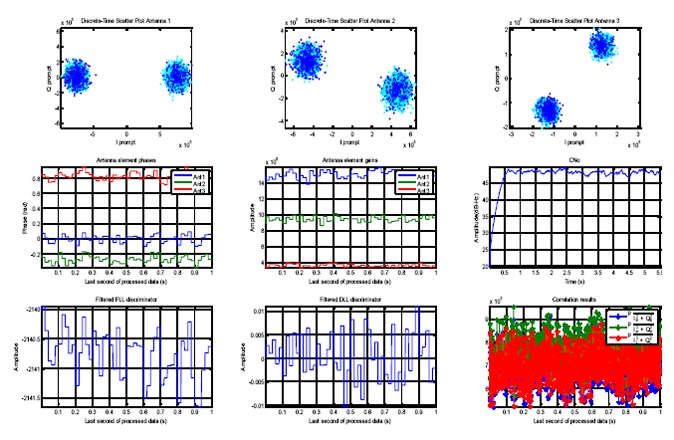
Tracking results of the multi-antenna receiver for one satellite signal.

An open source MATLAB-based single antenna software receiver in [18] was further developed for multi-antenna receiver and the tracking, acquisition and position solution parts of the original software were modified. As shown in Figure 2 showing the receiver structure, only the satellite signals received at the reference antenna are acquired, tracked and used for the positioning and the locally generated codes obtained from processing the signal of this antenna are used to measure the phases and amplitudes of the signals at the other antennas. Therefore, the estimated discriminator outputs at different antennas differ only on phases and amplitudes (changing only by changes in the signal DOA). For illustration, in Figure 5, the tracking results of the multi-antenna receiver for the signal of one satellite are shown. In this figure, the scatter plots and measured amplitudes and phases of antenna elements are shown. Outputs of phase lock loops (PLL) and delay lock loops (DLL), early, late and prompt correlation results and C/N_0_ variations are also shown.

**Figure 6.**
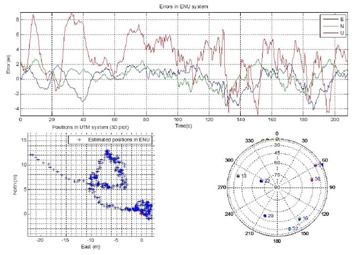
Estimated position solutions and the corresponding errors by the software receiver.

Figure 6 shows the position errors in the ENU coordinate system for the first 200 s of the calibration stage that includes 80 s in the static mode followed by two circular motions (each 60 s). The reference trajectory is obtained using NovAtel’s Inertial Explorer software. This figure also shows the satellites constellation at the time of data collection. The GPS satellite PRN number and their azimuths and elevation angles are presented in Table 1.

**Figure 7.**
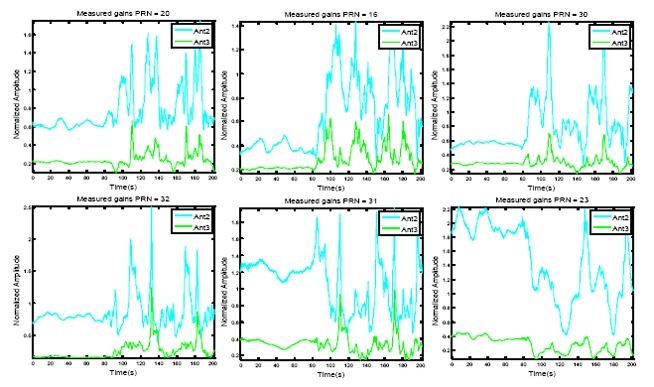
Measured amplitudes for Antenna 2 and Antenna 3 for PRNs 20, 16, 30, 32, 31 and 23.

**Figure 8.**
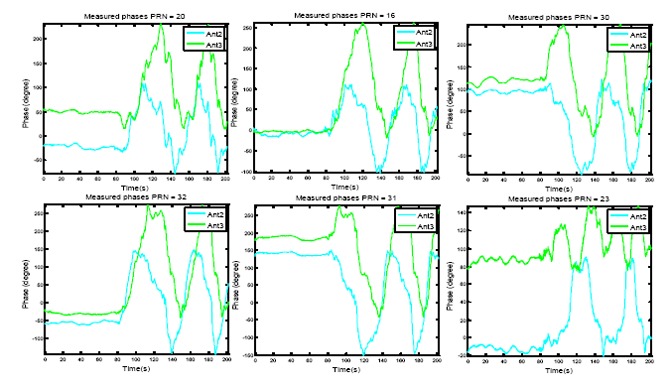
Measured phases for Antenna 2 and Antenna 3 for PRNs 20, 16, 30, 32, 31 and 23.

The measured amplitudes and phases for these satellite signals *versus* time are shown in Figures 7 and 8, respectively. In each figure, the measured values for Antenna 2 and 3, from the discriminator outputs after filtering, are shown (as mentioned before, the phase of reference antenna is kept almost zero and its amplitude is kept to 1). An exponential filter is used for filtering with a smoothing factor of *μ* = 0.05 as:

(18) $$\begin{array}{l} \left|{\text{z}}_{i}\right|=\mu \left|{\text{ỹ}}_{i}\right|+\left(1-\mu \right)\left|{\text{z}}_{i-1}\right|\\ ∠{\text{z}}_{i}=\mu ∠{\text{ỹ}}_{i}+\left(1-\mu \right)∠{\text{z}}_{i-1} \end{array}$$

in which, |**z***_i_*| and ∠**z***_i_* are the amplitude and phase of the filter output vector. Due to the normalization with respect to the reference antenna, Doppler frequency and its variations, which are precisely tracked by the receiver PLL, are removed from the measurements. These plots indicate that the measured amplitudes from each antenna not only are different but also vary by the changes in the signal DOA in the body frame coordinate. For example, the amplitude of the signal at Antenna 2 varies in the range of ±3*dB* with respect to the reference antenna. The plots in Figure 8 show that the measured relative phases also vary depending on the satellite signal DOA and the array orientation. For example, the signal received from the satellite with PRN number 23 which is the one with the highest elevation has the least variation in the circular motion and the signals of the satellites with low elevation angles show the highest variations.

**Figure 9.**
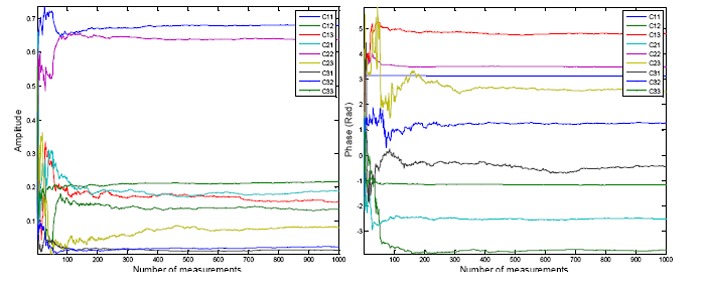
Amplitude and phase of the estimated calibration coefficients.

Figure 9 shows the amplitudes and phases of the estimated calibration coefficients from the first stage of the calibration process. The plots illustrate the estimated elements of the matrix **C̄** *versus* the number of measurements from the first minimization in Equation (12). The results show that after a few hundred measurements, the estimated coefficients do not change significantly.

**Figure 10.**
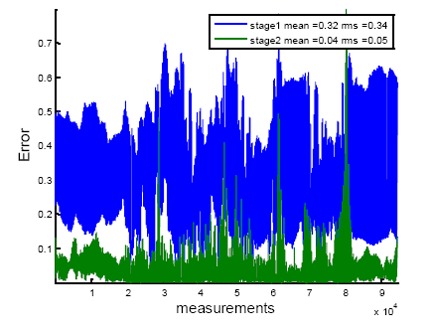
Normalized errors for two stages of the calibration process.

The estimated compensation amplitudes and phases from the second optimization in Equation (12) are stored in look up tables. For the grid, *g_A_* and *g_E_* are chosen as 360 and 18, respectively. This stage of calibration compensates for the differences in antennas gain and phase patterns, phase centres variations, antenna configuration perturbation, platform scattering and other parameters that depend on the signal DOA. In order to evaluate the performance of each stage of the calibration process, Figure 10 shows the normalized errors for these two stages, which are calculated using the following expressions:

(19) $$\begin{array}{lll} \text{stage}1) & \text{error}= & \frac{\left\|{\text{ỹ}}_{i}\delta^{T}\bar{\text{C}}{\text{a}}_{i}-\bar{\text{C}}{\text{a}}_{i}\right\|}{\left\|{\text{ỹ}}_{i}\delta^{T}\bar{\text{C}}{\text{a}}_{i}\right\|}\\ \text{stage}2) & \text{error}= & \frac{\left\|{\text{ỹ}}_{i}\delta^{T}\bar{\text{C}}\bar{\text{P}}(\Phi_{l},\Lambda_{k}){\text{a}}_{i}-\bar{\text{C}}\bar{\text{P}}(\Phi_{l},\Lambda_{k}){\text{a}}_{i}\right\|}{\left\|{\text{ỹ}}_{i}\delta^{T}\bar{\text{C}}\bar{\text{P}}(\Phi_{l},\Lambda_{k}){\text{a}}_{i}\right\|}\\ & & (\varphi_{i}^{B},\lambda_{i}^{B})\in (\Phi_{l},\Lambda_{k})\;l=1,\cdots ,g_{A},\;k=1,\cdots ,g_{E} \end{array}$$

### 4.3. Results for the Receiver Operation Mode

It was shown in Section 3.2 that the use of the proposed iterative method along with using the estimated **C̄** and **P̄**(Φ*_l_*, Λ*_k_*), leads to accurate estimates of satellite signal steering vectors in the receiver operation mode. This method provides estimates of satellite signal steering vectors based on the received signals and calibration results without any aiding information from the IMU. Estimating the steering vectors of the GNSS signals can be used in different multi-antenna applications such as enhancing SNR, anti-interference, multipath mitigation and attitude determination.

In this section, the calibrated three-element array is used in two practical applications to highlight the effectiveness of the proposed method in the receiver operation mode. In the first example, the heading angle of the moving vehicle is estimated from the signal of only one satellite and in the second example, using the signal of each satellite, the antenna array beam pattern is shaped to steer the main lobe of the beam pattern toward the direction of that signal and accordingly to enhance its carrier to noise ratio (C/N_0_).

#### 4.3.1. Attitude Determination

Precise attitude determination is essential in various applications. Differential carrier phase measurements from several receivers with precisely determined baselines can be employed to derive attitude parameters. Since performance degrades by shortening the baselines between receivers, the applicability of these methods might be confined to applications where, antennas separation is extremely limited. Herein, beamforming using a calibrated antenna array is employed for attitude determination, which can be an alternate option in these situations. Moreover, in contrast to standard attitude determination, in this method only one GNSS signal along with an adequate number of antenna elements is enough for attitude determination. This is another advantage of this approach, especially in challenging environments where the number of available signals is low. However, the challenge is carrier phase multipath.

In order to evaluate the performance of attitude determination based on beamforming, a test was performed with the calibrated antenna array. For the sake of comparison, the SPAN LCI system was used as a reference to evaluate the accuracy of the estimated heading angles. Assuming a horizontal motion and considering Equations (7) and (8), the heading angle can be estimated using the following relation:

(20) $${\text{e}}^{B}=\left[\begin{array}{ccc} \cos\left(y\right) & \sin\left(y\right) & 0\\ -\sin\left(y\right) & \cos\left(y\right) & 0\\ 0 & 0 & 1 \end{array}\right]{\text{e}}^{\text{ENU}}$$

where **e***^ENU^* and **e***^B^* are obtained from Equations (7) and (16), respectively.

Since the measurements employed in heading estimation are the same as those used for the calibration process, this test is more of a consistency test than an accuracy performance test.

**Figure 11.**
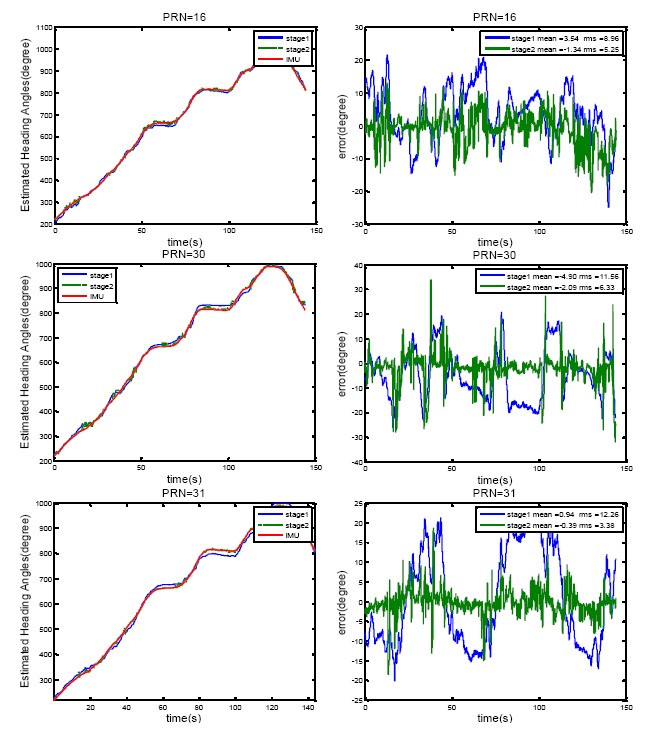
Heading angle determination results and the estimated errors compared to the IMU estimates for PRN 16, 30 and 31.

Figure 11 shows the results of heading determination for PRN 16, 30 and 31. In Figure 11, estimated heading angles for a moving vehicle for two cases are compared with the IMU derived heading angles. In the first case, only the first stage of the optimization method is employed (only constant uncertainty matrix C is considered) whereas in the second case look-up tables obtained from the second stage are also used for improving the heading estimation. Five iterations were adopted for the iterative method in order to estimate the steering vectors. Figure 11 also plots the root mean square (RMS) agreement with the IMU estimates. The precise calibration process improves the agreement by 3° to 9°. However, it is not the case for all PRNs. PRNs with the higher elevation angles show poorer accuracy for heading angle estimation such that the satellite located in zenith cannot be used to sense the horizontal motion. Moreover, the errors due to multipath and noise are higher for the signals of satellite with low elevation angles.

**Figure 12.**
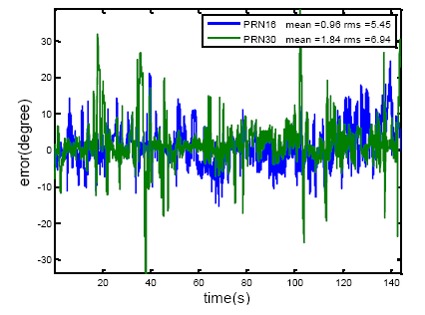
Consistency of heading estimates for different satellites (PRN 16 and 30 are compared to PRN 31).

In order to check the consistency between the estimated heading angles from these three PRNs, estimated heading angles from PRN 16 and 30 are subtracted from those of PRN 31. The results, along with the mean and the RMS values are shown in Figure 12. The results show a consistency of 5° to 7° between the estimated heading angles for these PRNs.

**Figure 13.**
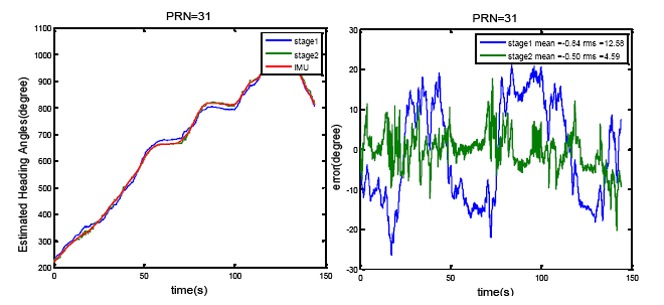
Heading angle determination results for PRN 31 when this PRN is not used in the calibration process.

In another test, in order to verify the calibration and estimated heading results independently, PRN 31 is excluded from the calibration process and stage 1 and 2 of the calibration method are repeated without using this PRN. Then the heading angles are estimated using the signal of this PRN and the calibrated array. The results for this test are shown in Figure 13. This figure shows a very small degradation compared to the results shown in Figure 11 for PRN 31.

#### 4.3.2. C/N_0_ Enhancement

In order to show the performance of C/N_0_ enhancement employing the calibrated array, the measured C/N_0_ values after beamforming for the array antenna and for the single antenna are compared. The C/N_0_ values are calculated as the ratio of the received satellite signal power to the noise density calculated by a noise floor estimator that correlates the received signal with a normalized fictitious PRN [19]. The minimum power distortionless response (MPDR) beamformer is employed to shape the array beam pattern [16]. In doing so for the signal of each satellite, the steering vector is estimated in the receiver operation mode and the corresponding optimal array weight vector **w** is obtained as:

(21) $$\text{w}\;={\hat{\text{R}}}^{-1}{\text{a}}_{i}{\left({\text{a}}_{i}^{H}{\hat{\text{R}}}^{-1}{\text{a}}_{i}\right)}^{-1}$$

where **R̂** is the estimated correlation matrix which is defined as:

(22) $$\hat{\text{R}}≜\frac{1}{K}\sum_{k=0}^{K-1}{\text{y}}_{k}{\text{y}}_{k}^{\text{H}}$$

**Figure 14.**
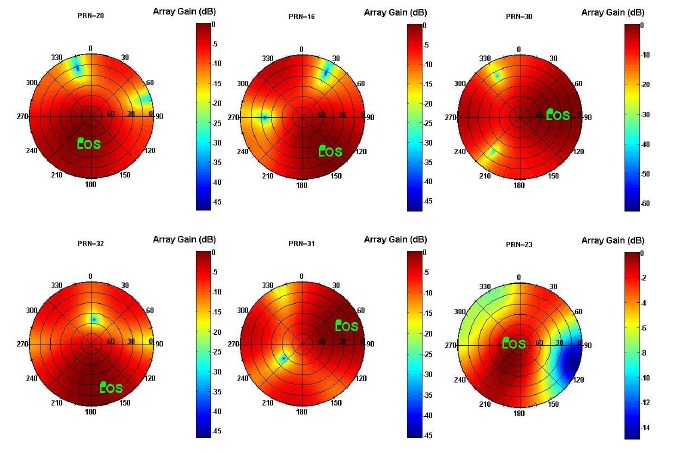
Normalized array antenna beam patterns after beamforming for PRNs 20, 16, 30, 32, 31 and 23.

**Figure 15.**
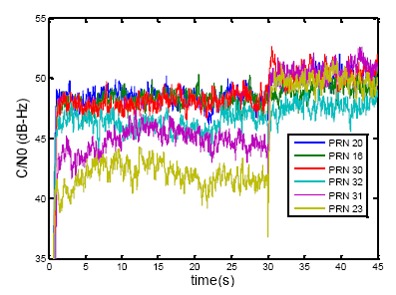
Comparing C/N_0_ for a single antenna receiver and the beamformer for PRNs 20, 16, 30, 32, 31 and 23.

The expression in Equation (22) is the estimation of the covariance matrix using *K* successive measurements. The MPDR beamformer maintains the main lobe of the antenna array beam pattern in the direction of the desired signal thereby minimizing the power of noise and interference. Figure 14 shows the normalized polar beam patterns *versus* azimuth and elevation angles for the satellite signals. It can be observed that for the signal of each PRN, the main lobe is steered toward the direction of that PRN. In order to have an actual sense of the improvement achieved by applying the beamforming process, the measured C/N_0_ values for the satellite signals at the reference antenna and the beamformer are illustrated in Figure 15 in which, after 30 s, the receiver switches from the single antenna structure to the multi-antenna structure. Due to the non-uniform antenna gain patterns and different amplifier gains, C/N_0_ values increase differently for signals coming from different directions.

## 5. Conclusions

A novel two-stage precise calibration method using satellite signals for GNSS applications was proposed. In this method, all of the main uncertainties in the calibration process were modeled and it was shown that they could be separated into two parts; one is constant and the other one consists of DOA-dependent parameters. One solution for the associated optimization to this problem was introduced in the form of two eigenvalue decomposition problems. Although the estimated constant term could provide coarse estimates of satellite steering vectors, for more precise calibration the second stage provided compensation amplitude and phase for each incident signal corresponding to its DOA in a form of a set of look up tables for each antenna element. The proposed two-stage calibration process not only simplified the interpretation of uncertainties, but also was used to come up with an iterative method with a fast convergence to accurately estimate satellite signal steering vectors. The effectiveness of this method in both calibration and receiver operation modes was evaluated by using a set of collected GPS signals in two different GNSS multi-antenna applications. Although an IMU unit was employed in this case for calibration and its performance evaluation, heading, roll and pitch angles could also have been provided by the carrier phase measurements obtained by the antenna array using standard GNSS attitude determination methods. In this paper, the measurement noise and carrier phase multipath were not explicitly considered in the mathematical model of the calibration although the measurements might be affected by them. This may reduce the accuracy of the calibration results. For example, although carrier phase multipath might be compensated in the second stage of the calibration process for certain observables, the compensation values are not necessarily applicable to the other observations. Moreover, a weighting model could be used to reduce the effect of noisier observations (e.g., signals from satellites with lower elevation angles) instead of using the equal weight model employed in this paper. Herein the results of calibration were verified through attitude determination and beamforming applications and employing the IMU measurements as a reference. However, estimating the accuracy of the calibration procedure as an independent block has not yet been investigated. Studying an approach to express the accuracy of the calibration results as a function of azimuth and elevation angles is suggested as future work. Furthermore, repeating the performed data collection for a certain antenna array for a long period of time in different environments and checking the consistency between the results to observe the effect of carrier phase multipath is suggested for further research on this paper.

## Figures and Tables

**Table 1.** Azimuth and elevation angles of satellites at the time of the data collection employed for the array calibration.

**PRN**	**20**	**16**	**30**	**32**	**31**	**23**
Azimuth (Degree)	204.1	146.6	78.3	163.7	58.8	292.2
Elevation (Degree)	52.1	37.5	45.2	29.1	28.9	68.6

## Appendix A

Considering Equation (5), the cost function in Equation (9) can be rewritten as:

(A.1) $$\begin{array}{l} =\underset{i}{\text{∑}}{\left\|{\text{ỹ}}_{i}\delta^{T}\text{CP}(\Phi_{l},\Lambda_{k}){\text{a}}_{i}e^{j\theta (t)}-\text{CP}(\Phi_{l},\Lambda_{k}){\text{a}}_{i}e^{j\theta (t)}\right\|}^{2}\\ =\underset{i}{\text{∑}}{\left\|{\text{ỹ}}_{i}\delta^{T}\text{CP}(\Phi_{l},\Lambda_{k}){\text{a}}_{i}-\text{CP}(\Phi_{l},\Lambda_{k}){\text{a}}_{i}\right\|}^{2}{\left\|e^{j\theta (t)}\right\|}^{2}\\ =\underset{i}{\text{∑}}{\left\|{\text{ỹ}}_{i}\delta^{T}\text{CP}(\Phi_{l},\Lambda_{k}){\text{a}}_{i}-\text{CP}(\Phi_{l},\Lambda_{k}){\text{a}}_{i}\right\|}^{2}\\ =\underset{i}{\text{∑}}{{\text{a}}_{i}}^{H}{\text{P}}^{H}(\Phi_{l},\Lambda_{k}){\text{C}}^{H}{\left({\text{ỹ}}_{i}\delta^{T}-{\text{I}}_{N}\right)}^{H}({\text{ỹ}}_{i}\delta^{T}-{\text{I}}_{N})\text{CP}(\Phi_{l},\Lambda_{k}){\text{a}}_{i} \end{array}$$

or equivalently as:

(A.2) $$=\text{vec}{\left(\text{CP}(\Phi_{l},\Lambda_{k})\right)}^{H}(\sum_{i}{{\text{A}}_{i}}^{H}{\left({\text{ỹ}}_{i}\delta^{T}-{\text{I}}_{N}\right)}^{H}({\text{ỹ}}_{i}\delta^{T}-{\text{I}}_{N}){\text{A}}_{i})\text{vec}(\text{CP}(\Phi_{l},\Lambda_{k}))$$

where **A***_i_* is defined in Equation (14) and the rank of each matrix in the inner summation of Equation (A.2) is *N* − 1. Ignoring the noise and gridding resolution effect, all of the matrices in the summation are the same, since for all of them the signals arrive from the same direction. Hence, the rank of matrix $\underset{i}{\text{∑}}{{\text{A}}_{i}}^{H}{\left({\text{ỹ}}_{i}\delta^{T}-{\text{I}}_{N}\right)}^{H}({\text{ỹ}}_{i}\delta^{T}-{\text{I}}_{N}){\text{A}}_{i}$ is *N* − 1. Singular value decomposition of this matrix can be expressed as:

(A.3) $$\sum_{i}{{\text{A}}_{i}}^{H}{\left({\text{ỹ}}_{i}\delta^{T}-{\text{I}}_{N}\right)}^{H}({\text{ỹ}}_{i}\delta^{T}-{\text{I}}_{N}){\text{A}}_{i}=\left[\begin{array}{cc} {\text{U}}_{s} & {\text{U}}_{n} \end{array}\right]\;\left[\begin{array}{cc} {\text{S}}_{s} & 0\\ 0 & {\text{S}}_{n} \end{array}\right]\;\left[\begin{array}{c} {\text{U}}_{s}^{H}\\ {\text{U}}_{n}^{H} \end{array}\right]$$

where **S***_S_* and **S***_n_* are the diagonal matrices of the eigenvalues corresponding to the signal and null spaces, respectively, and **U***_S_* and **U***_n_* are the matrices of the normalized eigenvectors corresponding to the signal and null spaces, respectively. Using this notation, the solution of *vec*(**CP**(Φ*_l_*,Λ*_k_*)) is in the null space and belongs to *SPAN*(**U***_n_*) with the dimension of *N*^2^ − (*N* − 1). Considering noise measurement and the gridding resolution, the dimension of the null-space (or noise space) becomes smaller; however there are still many solutions available for *vec*(**CP**(Φ*_l_*,Λ*_k_*)).
